# WHY ARE SOCIALLY ISOLATED OLDER ADULTS UNABLE TO ACCESS SERVICES IN JAPAN? A PRELIMINARY REVIEW OF FOUR FACTORS

**DOI:** 10.1093/geroni/igad104.2387

**Published:** 2023-12-21

**Authors:** Tomoko Ikeuchi, Kazunori Kikuchi, Yu Taniguchi, Cullen Hayashida, Kae Ito

**Affiliations:** Tokyo Metropolitan Institute for Geriatrics and Gerontology, Tokyo, Tokyo, Japan; Tokyo Metropolitan Institute for Geriatrics and Gerontology, Tokyo, Tokyo, Japan; National Institute for Environmental Studies, Tsukuba, Ibaraki, Japan; University of Hawaii, Honolulu, Hawaii, United States; Tokyo Metropolitan Institute for Geriatrics and Gerontology, Tokyo, Tokyo, Japan

## Abstract

The COVID-19 pandemic has made it difficult for health and social service agencies to provide the necessary support to community-based older adults. It is believed that the number of socially isolated older adults without the necessary support has increased, but the actual situation is unclear. In this study, we conducted a self-administered mail survey targeting all local municipal governments and social welfare councils throughout Japan from September to October 2022 to examine the actual situation. The response rate was 18.7% (682/3654). Nearly 52% of respondents reported an increase in the number of socially isolated older adults during the pandemic, and 43.6% reported that providing support services to them was difficult. We specifically asked about the difficulties encountered in providing support and coded the open-ended responses (n=275). Four primary themes were identified: (1) generalized refusal by isolated older adults (n=79); (2) difficulty in locating (n=72); (3) difficulty in face-to-face communication (n=40); and (4) difficulty in supporting (n=73). Among the reasons for the difficulty in supporting isolated older adults were their diverse needs and tendency to not recognize their need for support, which may be due to mental illness or dementia. Our findings suggest that a better attempt at classifying refusals among social isolates is a first step to improving the impact of social and health services intervention. Further research is needed to clarify the reasons for refusal of the support provided and to identify the specific types of support that isolated older adults require.

